# 45-year-old Male with Bilateral Lower Extremity Wounds, Swelling, and Rash

**DOI:** 10.5811/cpcem.2022.11.58813

**Published:** 2023-02-09

**Authors:** Christina M. Sajak, Kevin M. Semelrath, Laura J Bontempo, T. Andrew Windsor

**Affiliations:** * University of Maryland Medical Center, Baltimore, Maryland; † University of Maryland School of Medicine, Department of Emergency Medicine, Baltimore, Maryland

**Keywords:** rash, diphtheria, CPC

## Abstract

A 45-year-old male presented to the emergency department (ED) with bilateral lower extremity pain, swelling, and associated atypical rash in the setting of polysubstance use and unstable housing. Laboratory tests showed an elevated white blood cell count and inflammatory markers.

## CASE PRESENTATION (DR. SAJAK)

A 45-year-old male presented to the ED for evaluation with a chief complaint of bilateral lower extremity pain and swelling. The patient reported three to four days of an open wound to his right foot and one to two days of a gradually worsening wound to the right ankle. He reported a history of intravenous drug use (IVDU) and had most recently been injecting into his bilateral feet. He had no associated fevers or chills, but he did note a non-tender, non-pruritic rash that began in his bilateral feet and spread upward to the bilateral thighs over the course of a few days.

The patient had a history of methicillin-resistant *Staphylococcus aureus* (MRSA) bacteremia, osteomyelitis of the left great toe that required a partial ray amputation, depression, and a prior motor vehicle collision that resulted in a cerebrovascular accident due to carotid injury, as well as a traumatic brain injury, tracheostomy, and multiple facial reconstructive surgeries. The patient smoked a quarter of a pack of cigarettes per day and chronically used alcohol, as well as cocaine, heroin, and marijuana. At the time of his evaluation, he was experiencing homelessness. His medications included methadone, escitalopram, and quetiapine. He had no known drug allergies. A full review of systems was notable only for bilateral lower extremity pain as well as the rash, wound, and swelling noted in his history of present illness. Pertinent negatives included a lack of fever, chills, or other systemic symptoms.

The patient was alert and oriented to person, place, and time. His temperature on arrival was 36.6° Celsius, heart rate was 86 beats per minute (bpm), blood pressure of 154/94 millimeters of mercury, respiratory rate was 15 breaths per minute, and oxygen saturation was 99% on room air. He had a body mass index of 24.7 kilograms per meter squared. He appeared well-developed and well-nourished. He had sequela of facial reconstructive surgery. Mucous membranes were moist. He had a tracheostomy in the midline of his neck, which was patent without discharge or surrounding erythema. He had full cervical range of motion. His pupils were 3 millimeters equal, round, and reactive to light bilaterally, and extraocular movements were intact. His heart had a regular rate and rhythm with normal S1 and S2 without a murmur. He had normal and symmetric radial and dorsalis pedis pulses bilaterally. He was found to have trace pitting edema of the bilateral feet and ankles. He had a normal respiratory effort with clear, equal breath sounds. There was no chest wall deformity or tenderness. His abdomen was soft, non-tender, and non-distended with normal bowel sounds.

**Image 1:**
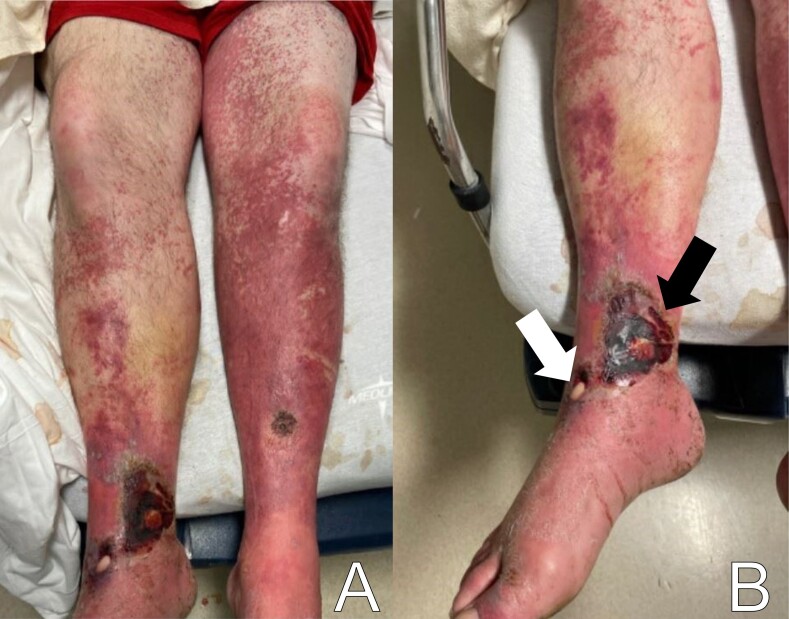
Photographs of a 45-year-old male with lower extremity wounds, swelling, and a rash. A) Bilateral lower extremities showing an erythematous, papular, non-blanching rash present from the anterior thighs to the feet. B) Right ankle ulcer with a membranous covering (white arrow) and wound with eschar (black arrow).

**Image 2:**
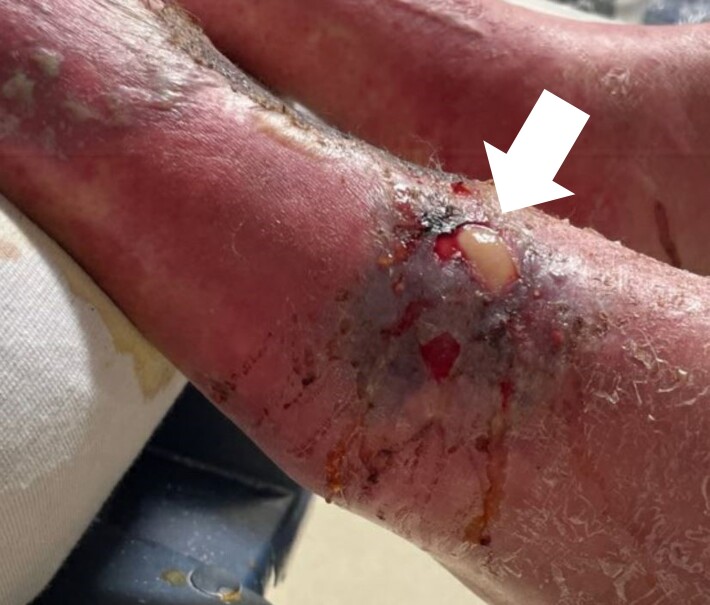
Photograph of an ulcer with membranous covering (white arrow) on the anterolateral right ankle of a 45-year-old male who presented with lower extremity wounds, swelling, and a rash.

Skin exam showed multiple open wounds to the bilateral lower extremities. He had an eschar present on the right ankle. There was a shallow ulcer present on the right ankle with a membranous covering. There was an erythematous, papular, non-blanching rash present from the anterior thighs to the feet (Images 1 and 2). The bilateral lower extremities had generalized tenderness to light touch. Scarring and erythema were noted on the left arm, which was slightly tender. No crepitus was palpable anywhere.

The patient had a contracture in his left upper extremity. All compartments of the bilateral lower extremities were soft. He had intact strength in his bilateral lower extremities, although he had some splinting secondary to pain. He was appropriate and cooperative.

**Image 3:**
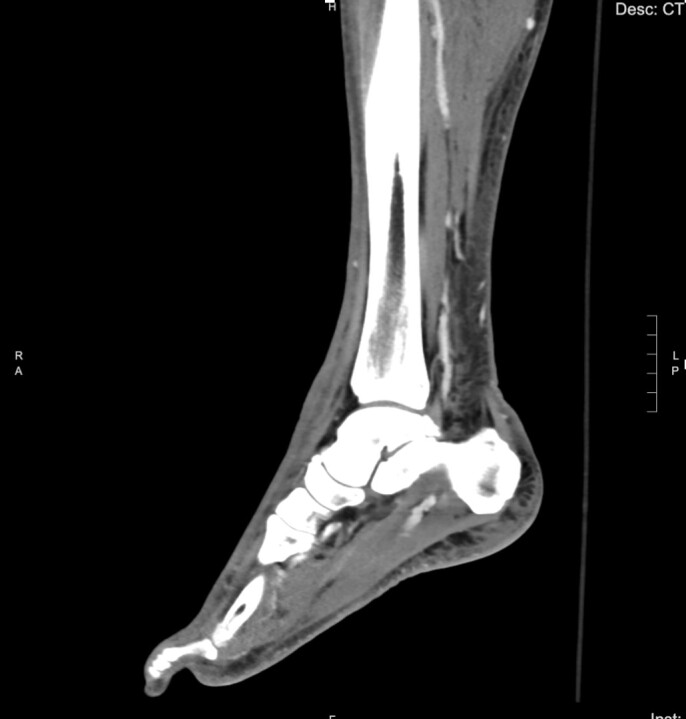
Computed tomography sagittal view of the distal right lower extremity of a 45-year-old male with lower extremity wounds, swelling, and a rash.

The patient’s initial laboratory studies are demonstrated in Table 1. The patient had radiographs taken of the bilateral lower extremities as well as a computed tomography (CT) of the right lower extremity. Radiology reads of the radiographs showed “postsurgical changes of both left and right fibular shaft resections with scattered surgical clips. No evidence of osteomyelitis. No acute fractures or dislocations. No evidence of subcutaneous gas.” Result of the CT of the right lower extremity demonstrated “extensive subcutaneous fat stranding, skin thickening with ulcerations consistent with cellulitis (Image 3). No rim-enhancing fluid collections to suggest an abscess. No evidence of soft tissue gas. Post-surgical changes of distal fibular shaft resection and multiple surgical clips. No significant osseous erosions or periosteal reaction.” The patient was given empiric antibiotic coverage with vancomycin and piperacillin-tazobactam due to concern for infection. He was given IV fluids, and morphine for pain control. He was admitted to the family medicine service where a diagnostic test was ordered, and the diagnosis was made.

## CASE DISCUSSION (DR. SEMELRATH)

When I first reviewed this case, it struck me that beyond the challenges of finding the correct diagnosis, this case also presents a challenge to some unconscious bias that we as emergency physicians may hold. The revelation of a history of substance abuse can lead to an initial assumption that the patient’s condition is a typical or expected consequence of their drug use. However, this case reveals that while a substance use disorder certainly does impact our patient’s health in a myriad of ways, it is of the utmost importance that we keep an open mind and an open differential diagnosis to avoid premature closure when presented with an unusual presentation.

To recap our patient’s presentation, this gentleman presented to the ED with several days of lower extremity pain and swelling, and a noticeable rash over the legs. His history revealed that the rash and swelling had developed over the course of several days, but that other than some pain in the legs, he had generally been feeling well without any systemic symptoms. Physical examination was that of a well-appearing man with vital signs remarkable only for mild hypertension. He had a normal heart rate and was afebrile. The examination of the lower extremities revealed an impressive-looking ulcerative lesion as well as a rash that extended up past his knees. The ulcer itself was noted to have a membranous covering on it, and the rash was non-blanching and flat. There was no crepitus noted, although when one looked at the representative pictures of the legs, they had edema present along the outline of the rash.

The radiologic studies that were obtained did not reveal an obvious diagnosis. The radiographs and CT show some soft tissue edema but no obvious localized abscess or subcutaneous emphysema. His laboratory studies revealed moderate leukocytosis with a bandemia, mild hyponatremia, and elevated serum inflammatory markers, namely the C-reactive protein (CRP) and the erythrocyte sedimentation rate (ESR).

When I approach a patient with a rash like this one, my first decision is whether the etiology is infectious or noninfectious. Here is where the unconscious bias can come into play. The patient was an active IV drug user; so the temptation is to attribute his presentation to the IV drug use. The immediate thought would be that this is a typical skin and soft tissue infection (SSTI) with an abscess that has been unroofed.

In terms of a “deadly differential” that emergency physicians should have in mind, necrotizing soft tissue infections such as necrotizing fasciitis or necrotizing myositis should always be high on the list. However, besides the visually impressive wounds being concerning for a necrotizing SSTI, the patient was not clinically toxic-appearing, had relatively normal vital signs, and was still alive after several days of his symptoms developing. These elements lower my pretest probability of a necrotizing SSTI. To diagnose necrotizing SSTI, there are a few hallmark features from the history and physical that should be present. It is typically rapidly developing, in most cases going from minor skin findings to life-threatening sepsis within 24 hours. It is also associated with severe pain, high fevers, a host of systemic symptoms, and the textbook finding of subcutaneous emphysema. All these findings were absent in this patient.

The laboratory findings in necrotizing SSTI, including a leukocytosis with a left shift of the differential, elevated inflammatory markers of ESR and CRP, and hyponatremia were present; however, given the nonspecific nature of these lab values, and the disconnect between the typical presentation of necrotizing SSTI and this patient’s presentation, I think I am on the wrong track here. So, when the clinical picture does not fit with your first few diagnoses, it’s time to widen your differential and look beyond any unconscious bias that may be in play.

Other infectious etiologies to consider are endocarditis with septic emboli, cellulitis, and syphilis. The patient’s IVDU puts him at risk for endocarditis with septic emboli. Skin trauma from IVDU and potentially poor hygiene related to homelessness put him at risk for cellulitis. Syphilis must be considered because although the patient did not specifically state that the rash began on his soles, he did report that the rash began on his feet without specifying which area. However, the patient was afebrile, had no pain, did not have a cardiac murmur, and was systemically well despite having symptoms for up to four days. Overall, his presentation is not consistent with a systemic or localized infection, thus lowering these choices on my differential diagnosis.

With a deep, violaceous rash, one of the things I always consider is some form of vasculitis. While the rash is consistent with a vasculitis, a bad vasculitis generally affects other organ systems, commonly the kidneys or lungs, as well. This patient had only a minimally elevated creatinine and had no pulmonary symptoms such as dyspnea or a cough, making vasculitis less likely to be the cause of his purpuric rash.

Other autoimmune processes must also be considered when a patient presents with a purpuric rash. Thrombotic thrombocytopenic purpura (TTP) is a reasonable concern given the ulcerative lesion and the associated rash. The classic pentad of TTP is thrombocytopenia, acute kidney injury, hemolytic anemia, fever, and major (e.g., seizure) or minor (e.g., headache) neurologic changes. However, the patient’s presentation does not fit with this very well, given his normal platelet level, very mildly elevated creatinine, normal hematocrit, and lack of fever or neurological complaints. While idiopathic thrombocytopenic purpura (ITP) is more common than TTP and can cause a purpuric rash in otherwise well-appearing patients, the lack of thrombocytopenia rules out this etiology. Henoch-Schönlein purpura could fit with the violaceous rash, but this is a pediatric disease, and the patient also does not have the associated findings of abdominal pain or renal impairment.

Another form of autoimmune disease that presents primarily with dermatologic findings is pyoderma gangrenosum (PG). Despite its name, this disease is neither pyogenic (pus forming) nor gangrenous (necrosis forming). It is a neutrophilic infiltrative dermatosis, where neutrophils locally invade the soft tissues, causing ulcerative lesions to form. Typically, multiple ulcerative lesions are present with at least one occurring on the anterior lower leg. There is an association of PG with other autoimmune diseases, particularly the inflammatory bowel diseases. There is also a strong association of PG and pathergy (being trauma-induced). We know that this patient had a substance use disorder and injected substances intravenously, which can be enough trauma to induce PG. Unfortunately, PG is typically a diagnosis of exclusion; so it would be difficult to make this diagnosis primarily from the ED.

Overall, the patient appeared too clinically well for a diagnosis of necrotizing SSTI to be a viable diagnosis and he had no known history that should put him at risk for autoimmune processes such as a vasculitis, TTP, ITP or PG. So, once again, I have to go back and widen my differential diagnosis. I know he was currently experiencing homelessness, so that puts him at risk for certain infectious diseases due to lack of hygienic services, poor nutrition, and exposure to the elements.

Looking back at the physical exam, one detail that I did not address is the presence of a membranous covering on the wound bed itself. Where else in medicine have I heard the term membranous covering? Diphtheria. While most of us are familiar with the typical presentation of diphtheria as a primary respiratory illness, there is also a cutaneous form of it. People who are at risk for contracting cutaneous diphtheria are those exposed to the bacteria in droplets and who are immunocompromised. Because the patient used IV drugs, he was at risk of having an undiagnosed immunocompromising condition.

Especially in this patient who was also experiencing homelessness, I have to look at the environment that the patient was occupying. The patient likely did not have ready access to medical care, most likely had a poor nutritional status, which can impair immune function, and his IV drug use could expose him to numerous bloodborne pathogens that can impact his ability to mount an adequate immune response. Additionally, many people experiencing homelessness will sleep near or on the grates of the public utilities because they are warm spots. However, this also means that the homeless person is continuously exposed to a warm, wet environment from steam escaping from the vents. This results in an essentially continuous droplet exposure.

To summarize this case, I have a patient who was clinically non-toxic but had wounds on his legs that appeared ulcerative with a membranous covering and an extensive, associated, violaceous rash. I believe the diagnostic test that should have been done is a wound culture, which would confirm a diagnosis of cutaneous diphtheria.

## CASE OUTCOME (DR. SAJAK)

The diagnostic test obtained in this case was a wound culture. The patient was taken to the operating room by the surgical consulting team and had intraoperative biopsies and cultures that demonstrated growth of *Corynebacterium diphtheriae*, and the diagnosis of cutaneous diphtheria was made. His blood cultures also grew methicillin-sensitive *S. aureus* (MSSA), *Streptococcus pyogenes*, and coagulase negative staphylococcus. He had an echocardiogram that did not show evidence of endocarditis. Infectious disease was consulted, and the patient ultimately received three days of vancomycin, piperacillin-tazobactam, and clindamycin before switching to oxacillin for the remainder of his hospital course. He was discharged to a subacute rehabilitation facility on oral cephalexin for a total duration of antibiotic therapy of 16 days.

## RESIDENT DISCUSSION (DR. SAJAK)

*C. diphtheriae* is an aerobic, gram positive bacillus that was first described by Hippocrates as early as the fifth century before the common era.^1^ By the end of the 19^th^ century, the bacterium had been observed and cultivated by two separate scientists, Edwin Klebs and Fredrich Loffler. It was thereafter discovered that the bacterium produces a toxin when lysogenized by corynebacteriophages, and the toxin is responsible for the clinical presentation of diphtheria. The mechanism of action of the toxin is through the inactivation of elongation factor 2 (EF-2), thereby inhibiting protein synthesis.^2^

Clinical infection with *C. diphtheriae* has a typical incubation period of two to five days, followed by an infectious period of two to six weeks without antibiotic treatment.^1^ Infection can occur on any mucous membrane and is classified by the location of the infection. Cutaneous diphtheria, as described in this patient, occurs in the skin and can also be accompanied by a scaling rash or ulcers, which are typically well demarcated. Cutaneous diphtheria is associated with a lesser risk of systemic complications when compared to respiratory diphtheria.^1^

As the name suggests, respiratory diphtheria occurs when *C. diphtheriae* infection occurs in the respiratory tract, in the nasal cavity, pharynx, tonsils, or larynx.^1^ Respiratory diphtheria can present with a constellation of symptoms including pharyngitis, dysphagia, fever, cervical lymphadenopathy, and epistaxis. Pseudomembranes can also be seen at the site of clinical infection with respiratory diphtheria, often appear gray in color, and are composed of fibrin, bacteria, and inflammatory cells.^2^ Respiratory diphtheria can be associated with airway compromise from growth of a pseudomembrane in the trachea and/or sloughing of pieces of a pseudomembrane into the airway, as well as systemic complications such as myocarditis, polyneuropathy, paralysis, and renal failure. Mortality of respiratory infections is approximately 5–10%, even with appropriate treatment.^1^

*C. diphtheriae* infections are seen with higher frequency in tropical climates and developing countries. They are more prominent in the unvaccinated and immunocompromised populations. However, there are also reports of asymptomatic nasopharyngeal carriers.^2^ Infection is known to occur through droplets, fomites, and open wounds.^3^ Diagnosis is often made via culture, such as in the case of this patient; however, there is also an immunogenicity test, called the modified Elek test, that is run at the Pertussis and Diphtheria Laboratory of the Centers for Disease Control and Prevention (CDC). This is an immunoprecipitation assay that tests for the toxigenicity of the strain of isolated *C. diphtheriae*.^4^

As it is an acute bacterial illness, antibiotics are indicated for both respiratory and cutaneous diphtheria. The CDC recommends initiation of penicillin or erythromycin.^4^ Typically patients are considered no longer infectious after 48 hours of antibiotic therapy but should have cultures drawn every 24 hours after completion of treatment until two consecutive negative results are obtained.^1^ For suspected cases of respiratory diphtheria, there is also an antitoxin that is given in consultation with the CDC. Importantly, this is not approved by the United States Food and Drug Administration but is available for use under the label of an investigational new drug and requires approval from the CDC diphtheria duty officer.^5^

It is important to note that active infection with *C. diphtheriae* may not confer immunity. Rather, prevention of diphtheria infection is accomplished through a toxoid vaccine, developed in the 1920s and later combined with the tetanus toxoid and the pertussis vaccine in the 1940s. In the United States, it is recommended that this combination vaccine be given as a four-shot series to children starting at age four months and concluding by age six years. Boosters are recommended at age 11–12 years and with every pregnancy. Boosters can also be given every 10 years for individuals at elevated risk, such as healthcare workers.^6^

## FINAL DIAGNOSIS

Cutaneous diphtheria

## KEY TEACHING POINTS

- Unconscious bias can limit our diagnostic thought process. To limit its effect, try to identify whether assumptions are being made about the patient that are not supported by evidence and then go back and widen your differential without those assumptions.
- Ulcers on the skin are most commonly a primary infectious process of the skin and soft tissue, but there are many other disease processes that have dermatologic manifestations as well.
- Diphtheria has a cutaneous form, which presents as ulcerative lesions with a membranous covering.
- Those at risk for cutaneous diphtheria are people who are unvaccinated against the disease, immunocompromised, have recently travelled, or have had prolonged exposures to warm, wet environments.

## Figures and Tables

**Table 1:** Laboratory values of a 45 year-old male with bilateral lower extremity wounds, swelling and rash.

Test name	Patient value	Reference range
Complete blood count		
White blood cell	15.5	4.5 – 11 K/mcL
Hemoglobin	13.6	11.9 – 15.7 g/dL
Hematocrit	39.9	35.0 – 45.0%
Platelets	341	153 – 367 K/mcL
Complete metabolic panel		
Sodium	133	136 – 145 mmol/L
Potassium	3.4	3.5 – 5.1 mmol/L
Chloride	99	98 – 107 mmol/L
Bicarbonate	25	21 – 30 mmol/L
Blood urea nitrogen	30	7 – 17 mg/dL
Creatinine	1.14	0.52 – 1.04 mg/dL
Glucose	116	70 – 100 mg/dL
Albumin	3.5	3.2 – 4.6 g/dL
Total bilirubin	1.1	0.3 – 1.2 mg/dL
Aspartate aminotransferase	30	14 – 36 units/L
Alanine aminotransferase	23	0 – 34 units/L
Alkaline phosphatase	71	38 – 126 units/L
Additional labs		
C-reactive protein	36.9	≤1.0 mg/dL
Erythrocyte sedimentation rate	61	0 – 25 mm/Hr

*dL*, deciliter; *g*, grams; *Hr*, hour; *K*, thousands; *mcL*, microliter; *mg*, milligram; *mm*, millimeter; *mmol*, millimole; *L*, liter.
